# Aptamer targeting EGFRvIII mutant hampers its constitutive autophosphorylation and affects migration, invasion and proliferation of glioblastoma cells

**DOI:** 10.18632/oncotarget.6066

**Published:** 2015-10-10

**Authors:** Simona Camorani, Elvira Crescenzi, David Colecchia, Andrea Carpentieri, Angela Amoresano, Monica Fedele, Mario Chiariello, Laura Cerchia

**Affiliations:** ^1^ Istituto per l'Endocrinologia e l'Oncologia Sperimentale “G. Salvatore” (IEOS), Consiglio Nazionale delle Ricerche (CNR), Naples, Italy; ^2^ Istituto Toscano Tumori (ITT), Core Research Laboratory (CRL) and Consiglio Nazionale delle Ricerche (CNR), Istituto di Fisiologia Clinica (IFC), Siena, Italy; ^3^ Dipartimento di Scienze Chimiche, Università degli Studi di Napoli “Federico II”, Naples, Italy

**Keywords:** aptamer, EGFRvIII, glioblastoma, PDGFRβ, combined treatment

## Abstract

Glioblastoma Multiforme (GBM) is the most common and aggressive human brain tumor, associated with very poor survival despite surgery, radiotherapy and chemotherapy.

The epidermal growth factor receptor (EGFR) and the platelet-derived growth factor receptor β (PDGFRβ) are hallmarks in GBM with driving roles in tumor progression. In approximately half of the tumors with amplified EGFR, the EGFRvIII truncated extracellular mutant is detected. EGFRvIII does not bind ligands, is highly oncogenic and its expression confers resistance to EGFR tyrosine kinase inhibitors (TKIs). It has been demonstrated that EGFRvIII-dependent cancers may escape targeted therapy by developing dependence on PDGFRβ signaling, thus providing a strong rationale for combination therapy aimed at blocking both EGFRvIII and PDGFRβ signaling.

We have recently generated two nuclease resistant RNA aptamers, CL4 and Gint4.T, as high affinity ligands and inhibitors of the human wild-type EGFR (EGFRwt) and PDGFRβ, respectively.

Herein, by different approaches, we demonstrate that CL4 aptamer binds to the EGFRvIII mutant even though it lacks most of the extracellular domain. As a consequence of binding, the aptamer inhibits EGFRvIII autophosphorylation and downstream signaling pathways, thus affecting migration, invasion and proliferation of EGFRvIII-expressing GBM cell lines.

Further, we show that targeting EGFRvIII by CL4, as well as by EGFR-TKIs, erlotinib and gefitinib, causes upregulation of PDGFRβ. Importantly, CL4 and gefitinib cooperate with the anti-PDGFRβ Gint4.T aptamer in inhibiting cell proliferation.

The proposed aptamer-based strategy could have impact on targeted molecular cancer therapies and may result in progresses against GBMs.

## INTRODUCTION

Glioblastoma multiforme (GBM) is a malignant and lethal form of brain cancer. The current standard for treatment of GBM consists of maximal surgical resection followed by radiotherapy with concomitant temozolomide, which frequently produces adverse effects, limiting therapeutic dosage increase. Despite treatment, the median survival for GBM patients remains less than 1.5 years [1]. Therefore, there is a pressing need to identify novel methods to more efficiently treat these tumors.

Epidermal growth factor receptor (EGFR) is a potent driver of GBM. EGFR gene amplification and overexpression occur in about 50% of primary GBMs and approximately half of tumors with amplification of the wild-type EGFR (EGFRwt) expresses the oncogenic mutant EGFR variant III (EGFRvIII, EGFR type III, de2-7 or ΔEGFR) [2, 3]. The overexpression of EGFRvIII with EGFR amplification is suggested as the strongest predictor of poor survival in GBMs [4-6]. EGFRvIII is generated by an in-frame deletion of 801 bp of coding sequence from exons 2 to 7 that removes amino acids 6 to 273 in the extracellular region of the EGFRwt. Thus, compared to EGFRwt, the truncated EGFRvIII cannot bind any known EGFR ligands but constitutively signals to downstream effector molecules [7].

EGFRvIII greatly enhances GBM tumorigenicity *in vivo* [8, 9] and stimulates cell invasion *in vitro* and *in vivo* [10, 11]. Different mechanisms of cooperation between EGFRwt and EGFRvIII have been reported, promoting malignant progression [12-15] and suggesting combinatorial targeting of both EGFR species. Regrettably, the results have so far been unsatisfactory in clinic given the high resistance of GBM to first-generation EGFR inhibitors, including erlotinib and gefitinib tyrosine kinase inhibitors (TKIs) and, to date, there is little evidence to sustain the use of such inhibitors as monotherapy [16-18]. One emerging cause that dictates GBM escape from EGFR-targeted therapies is the occurrence of alternative kinase signaling pathways that compensate the pharmacological perturbations. It has been recently shown that inhibition of EGFRvIII in GBM leads to increase of platelet-derived growth factor receptor β (PDGFRβ) expression and signaling as a growth rescue mechanism [19, 20], providing the rationale for co-inhibition of these receptors.

We generated a nuclease resistant 2′F-Pyrimidines (2′F-Py)-containing RNA aptamer, named CL4, as a high affinity (Kd: 10 nmol/l) ligand of human EGFR [21]. The aptamer specifically binds to the extracellular domain of the wild-type receptor thus inhibiting ligand-dependent EGFR autophosphorylation and downstream signaling pathways [21, 22].

Herein, we demonstrate that CL4 aptamer binds to the EGFRvIII mutant despite the deletion. Importantly, it inhibits EGFRvIII activation and constitutive signaling, thus interfering with migration, invasion and growth of GBM cells. We show that targeting EGFRvIII by CL4 causes upregulation of PDGFRβ and that CL4 and gefitinib cooperate with a validated anti-PDGFRβ aptamer [22] in inhibiting EGFRvIII-positive GBM cells growth.

Our results strongly encourage further *in vivo* investigation for aptamer-based approaches aimed at developing new therapeutics for GBM and other cancer types that depend on EGFRvIII and PDGFRβ for survival and growth.

## RESULTS

### CL4 binds to EGFRvIII mutant on cell surface

CL4 aptamer is a 39-mer 2′F-Py RNA that binds at high affinity to the extracellular domain of human EGFRwt both if expressed on cancer cells and in a soluble, recombinant form [21, 22]. Being EGFRvIII mutant a very appealing target for GBM treatment, here we investigated whether CL4 binds to EGFRvIII, even though the mutant receptor lacks most of domains I and II in the extracellular part of the protein.

**Figure 1:**
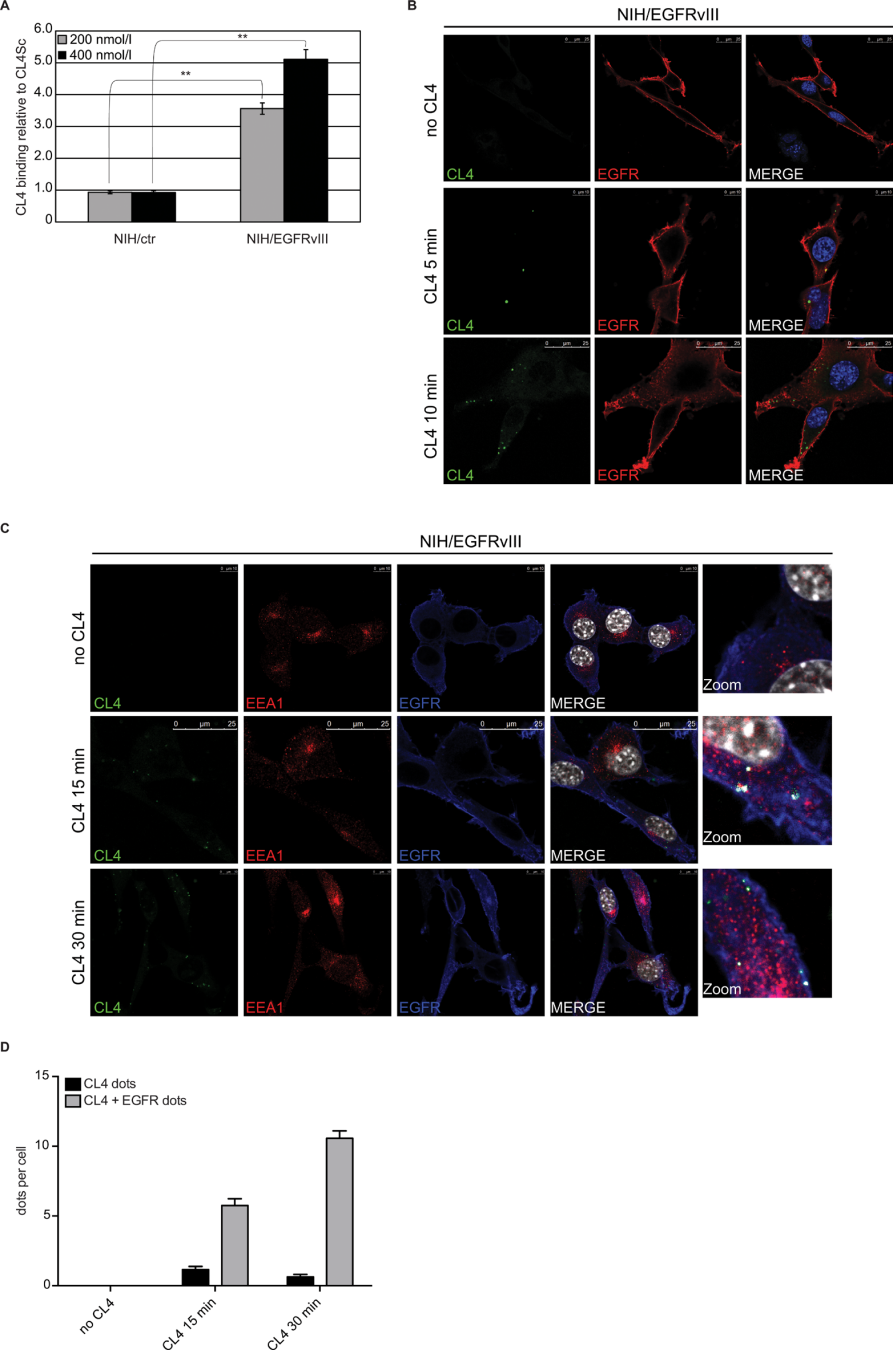
CL4 binds and internalizes into NIH/EGFRvIII cells **A.** Binding of 200 and 400 nmol/l CL4 to the indicated cells was detected by RT-qPCR. Results are expressed relative to the background binding detected with CL4Sc used as a negative control. Bars depict means ± SD (*n* = 3). **P < 0.01. **B.** NIH/EGFRvIII cells were incubated with 2.5 μmol/l FAM-labelled CL4 for 5 and 10 minutes. Cells were fixed and labelled with anti-EGFR antibody without permeabilization. CL4, EGFRvIII and nuclei are visualized in green, red and blue, respectively. **C.** and **D.** NIH/EGFRvIII cells were incubated with 2.5 μmol/l FAM-labelled CL4 for 15 and 30 minutes. Cells were fixed, permeabilized and labelled with anti-EGFR and anti-EEA1 antibodies. In C., CL4, EEA1, EGFRvIII and nuclei are visualized in green, red, blue and grey, respectively. **D.** Total CL4 dots per cell were counted in each frame of Z-stacks, then EGFRvIII signal was evaluated for each CL4 dot (signal lower than 20% was considered negative for colocalization while higher than 20% was considered positive).

Mouse NIH3T3 fibroblast cells, which show little to no expression of endogenous EGFRwt [15, 23], were engineered to overexpress human EGFRvIII (NIH/EGFRvIII) (supplementary Figure S1, left) and used as a testing platform for CL4 specificity. We first applied reverse transcription quantitative polymerase chain reaction (RT-qPCR) methods to detect cell binding of the aptamer. As shown (Figure 1A), CL4 bound, in a dose dependent manner, to NIH/EGFRvIII whereas it did not bind to cells transfected with empty vector (NIH/ctr). Results are expressed relatively to the background binding detected with a scrambled sequence (CL4Sc), used as a negative control. Next, we analyzed the binding of the fluorescent FAM-labelled CL4 to EGFRvIII on the surface of unpermeabilized cells, by confocal microscopy. As shown in Figure 1B and supplementary Figure S2A, CL4 aptamer localizes at membrane level of NIH/EGFRvIII, showing puncta of colocalization with EGFRvIII after only 5 minutes incubation whereas multiple CL4 dots were accumulated in the cytoplasmic side of cell membrane in 10 minutes incubation. Aptamer binding seems to be highly specific for NIH/EGFRvIII and very little to no signal for CL4 was revealed on NIH/ctr cells (supplementary Figure S2B). Furthermore, the uptake mechanism for anti-EGFR aptamer was investigated. To this aim NIH/EGFRvIII cells were incubated with CL4 aptamer for 15 and 30 minutes and then fixed, permeabilized and labelled with anti-EGFR and anti-EEA1 antibodies. As shown in Figure 1C and 1D, the aptamer colocalizes with EGFRvIII inside the cells. Further, active internalization of CL4 aptamer occurred by endosome recycling pathway [24] as demonstrated by the colocalization of CL4 EGFRvIII-bound with early endosome antigen 1 (EEA1), the main endosome marker (Figure 1C and supplementary Figure S3A). Only a very low CL4-signal was observed in NIH/ctr cells (supplementary Figure S3B).

**Figure 2:**
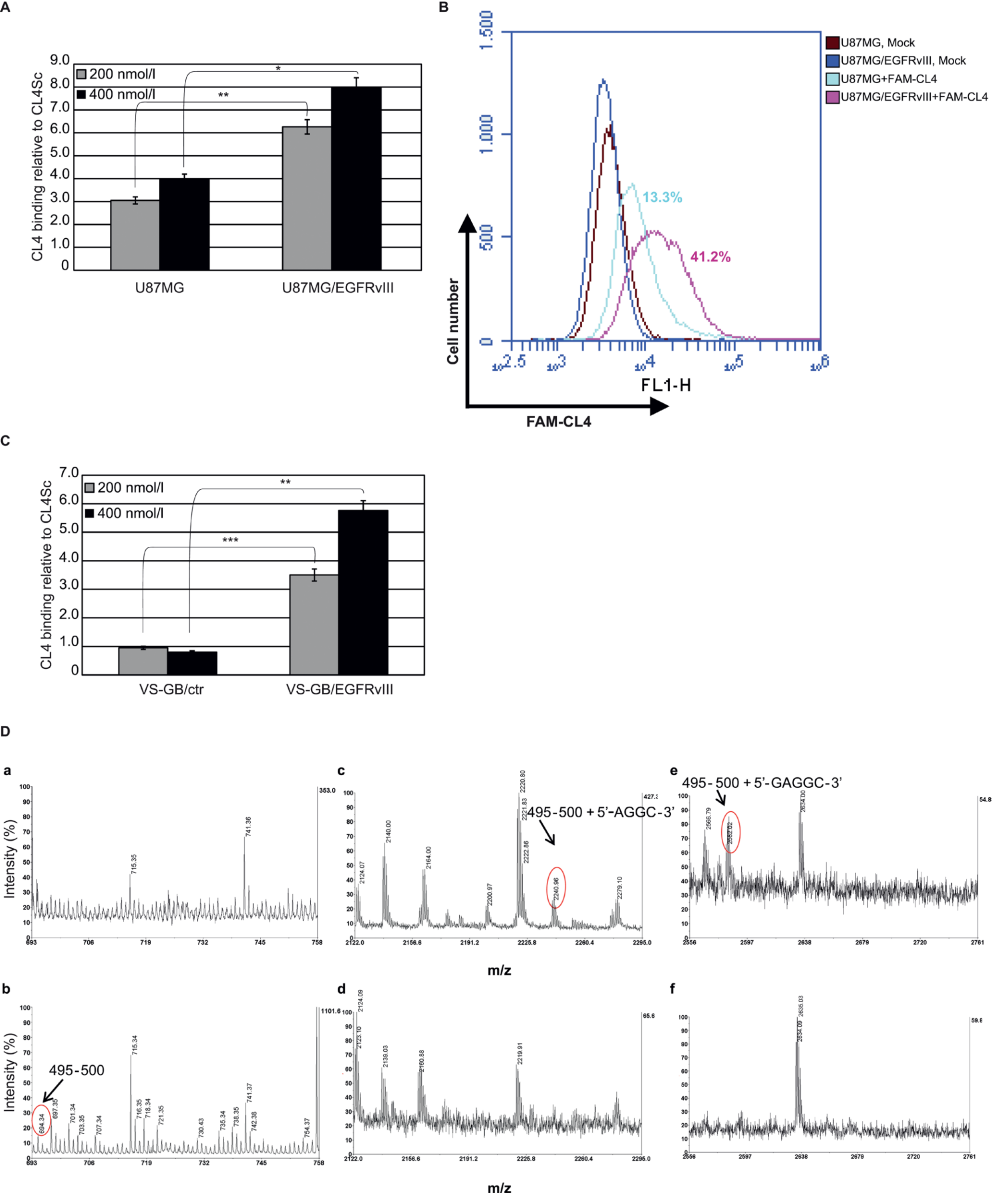
CL4 specifically interacts with EGFRvIII Binding of CL4 to the indicated cell lines was detected by (**A** and **C**) RT-qPCR or by **B.** flow cytometry. In **A** and **C**, results are reported as in the legend to Figure 1A. Bars depict means ± SD of three independent experiments. ***P < 0.001; **P < 0.01; *P < 0.05. In B, the percentages of cells that are positive for FAM-labelled CL4 binding as compared to background levels, detected with mock-treated cells, are reported. **D.** Partial MALDI-MS spectra of the cross-linked product (a, c, e) and of the control isolated protein (b, d, f) digested with trypsin and T1 ribonuclease. The attribution of the cross-linked peptides is shown.

As *in vitro* GBM models to detect CL4 binding to EGFRvIII, we used the highly tumorigenic U87MG cells and a primary GBM cell line, both stably engineered to express EGFRvIII (U87MG/EGFRvIII and VS-GB/EGFRvIII, respectively) since cultures established from tumors when grown *in vitro* lose EGFRvIII expression, as reviewed in [17]. Differently from U87MG cells, the VS-GB cell line does not express endogenously EGFRwt mRNA (supplementary Figure S1, right) and protein (supplementary Figure S1, left) at detectable levels. Accordingly to the above findings, CL4 bound to U87MG/EGFRvIII, as assessed by both RT-qPCR (Figure 2A) and flow cytometry (Figure 2B), at a higher extent with respect to parental U87MG cells. Further, it recognized VS-GB/EGFRvIII cells but not control cells (Figure 2C), thus confirming its specificity for EGFRvIII also in the absence of EGFRwt.

Thus, in an attempt to identify key amino acids of the receptor engaged in direct interactions with the aptamer, photo-chemical cross-linking was induced between the EGFRwt protein and the CL4 and then analyzed by a mass spectrometry (MS)-based approach. Briefly, the aptamer was combined in a 1:1 molar ratio with the extracellular domain of human EGFRwt in a soluble form and irradiated with UV light at 254 nm. The cross-linked products were digested with trypsin and the peptide mixture was then submitted to enzymatic hydrolysis with T1 ribonuclease to digest the polynucleotide chain. The resulting mixture was directly analyzed by matrix-assisted laser-desorption ionization (MALDI)-MS to identify the amino acid residues involved in the linkage with the CL4 aptamer. Figure 2D shows the MALDI spectra obtained. Most of the signals were assigned to EGFR fragments on the basis of their molecular mass and the specificity of the enzyme. It is worth noting that MALDI spectrum in the absence of the aptamer showed the occurrence of *m/z* 694.34, corresponding to peptide 495-500 (b). The same signal disappeared after cross-linking reaction (a) and two new signals occurred at *m*/*z* 2240.96 and 2582.02 that could not be assigned to any fragment within the protein sequence and were thus candidates for intermolecular cross-linked fragments (compare c and e to d and f). On the basis of the EGFR sequence and ribonuclease T1 digestions products, the two signals were identified as peptide 495-500, lying in the domain IV, linked to AGGC and GAGGC nucleotides, respectively. These results are in good agreement with the CL4 ability to bind to the EGFRvIII mutant given that the domain IV of EGFRwt is still preserved in the EGFRvIII mutant despite the deletion event.

### CL4 interferes with constitutive EGFRvIII activation and downstream signaling

**Figure 3:**
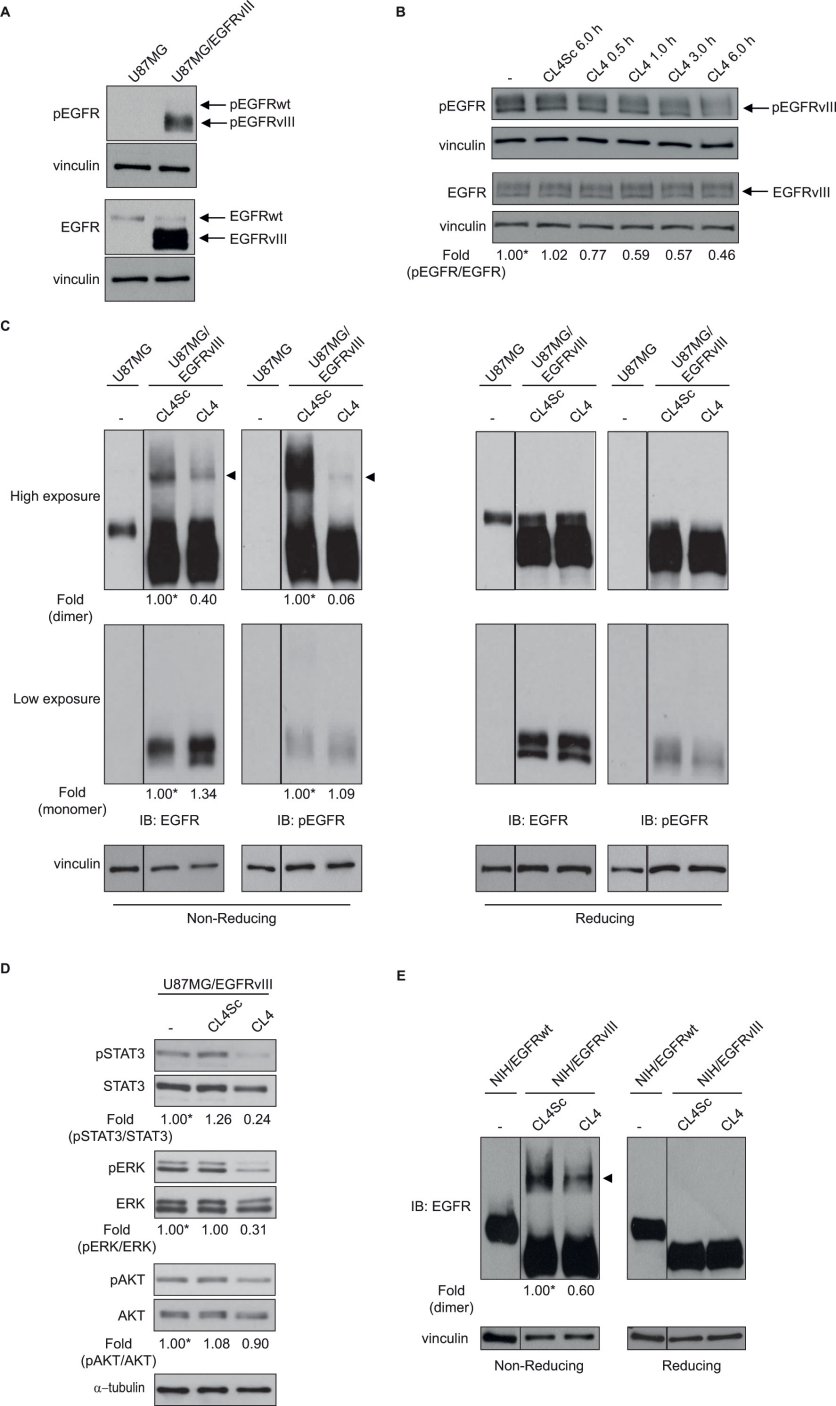
CL4 hampers EGFRvIII activation Lysates from **A.** U87MG and U87MG/EGFRvIII cells maintained in 2% FBS-containing medium for 6 hours or from **B.** U87MG/EGFRvIII cells maintained in 2% FBS-containing medium for 6 hours and then left untreated for further 6 hours or treated with 200 nmol/l CL4 or CL4Sc for the indicated times, were immunoblotted with anti-pEGFR and anti-EGFR antibodies, as indicated. In B, values below the blot indicate the ratio of pEGFR to total EGFR signal levels, normalized to the respective vinculin signal level, and reported as relative to untreated cells, arbitrarily set to 1 (labeled with asterisk). **C.** Equal amounts of lysates from U87MG or U87MG/EGFRvIII cells, maintained in 2% FBS for 6 hours and then treated with 200 nmol/l CL4 or CL4Sc for 24 hours, were run on 6% SDS-PAGE under non-reducing (left) and reducing (right) conditions and immunoblotted with anti-EGFR and anti-pEGFR antibodies. **D.** Lysates from U87MG/EGFRvIII cells treated as in “C” were immunoblotted with anti-pSTAT3, anti-pERK and anti-pAKT antibodies, as indicated. Filters were stripped and reprobed with anti-STAT3, anti-ERK and anti-AKT antibodies. **E.** Lysates from NIH/EGFRwt or NIH/EGFRvIII cells treated as in “C” were immunoblotted with anti-EGFR antibody under non-reducing (left) and reducing (right) conditions. In **C** and **E**, the band compatible with dimeric EGFRvIII species is depicted by the arrowhead. Equal loading was confirmed by immunoblot with anti-vinculin **(A-C, E)** or anti-α-tubulin antibody **D.**. **(C-E)** Values below the blots indicate signal levels relative to each controls, arbitrarily set to 1 (labeled with asterisk).

As a next step, we asked whether CL4 interferes with the autophosphorylation activity of EGFRvIII in U87MG/EGFRvIII cells. To minimize EGFRwt basal (unstimulated) phosphorylation, cells were maintained in low (2%) serum concentration for 6 hours before aptamer treatment. In these conditions, EGFRvIII was constitutively phosphorylated while basal activation/tyrosine phosphorylation of EGFRwt was undetectable (Figure 3A). As shown in Figure 3B, CL4 treatment (200 nmol/l) of U87MG/EGFRvIII cells attenuated activation of EGFRvIII reaching about 50% inhibition of phosphorylation following 6 hours-incubation, where it did not affect the total level of the EGFRvIII protein. No further increase in the inhibitory effect was observed by increasing either the aptamer concentration or the incubation time (not shown). Total and phospho-EGFRvIII levels were unaffected by CL4Sc negative control.

It has been reported that EGFRvIII mutant forms constitutively active receptor homodimers [25, 26] that are stabilized by intermolecular-disulfide bonds through free cysteine residues resulting as a consequence of the deletion event [27, 28]. Moreover, forced dimerization of EGFRvIII by using genetic strategies enhances receptor phosphorylation and downstream signaling, leading to increased GBM cell proliferation [29]. Thus, we asked whether the observed CL4-dependent reduction of EGFRvIII phosphorylation might be due to the aptamer's ability to hamper the formation of phosphorylated disulfide-bonded EGFRvIII complexes. To this aim, extracts from U87MG/EGFRvIII cells treated with CL4 or CL4Sc were run on sodium dodecyl sulfate polyacrylamide gel electrophoresis (SDS-PAGE) under non-reducing or reducing condition and blotted with anti-EGFR and anti-phospho-EGFR antibodies. As shown in Figure 3C (IB: EGFR, high exposure), a band compatible with the EGFRvIII dimer was clearly observed under non-reducing condition (left, arrowhead) which disappeared under reducing condition (right). The immunoblot with anti-phospho-EGFR antibodies revealed that this species was strongly phosphorylated. Remarkably, treating the cells with CL4 caused a drastic reduction of such phosphorylation. Under these conditions, no detectable disulfide-bonded EGFRwt homodimer was observed, as assessed by comparing the electrophoretic profile of lysates from U87MG/EGFRvIII and parental U87MG cells. Further, a substantial reduction of phosphorylation of EGFRvIII downstream effectors signal transducer and activator of transcription 3 (STAT3) and extracellular signal-regulated kinase (ERK) 1/2 was observed upon CL4 treatment of the cells (Figure 3D), suggesting a critical role of EGFRvIII dimerization for signaling. Conversely, no significant reduction was obtained on AKT activity, consistent with the lack of regulation of the phosphoinositide 3-kinase (PI3K) pathway in U87MG cells that harbor an inactivating mutation of the phosphatase and tensin homolog (PTEN) gene [30-32].

By using the same experimental conditions as for U87MG/EGFRvIII cells, we detected, also in NIH/EGFRvIII cells, higher-order EGFRvIII complexes that were significantly reduced in the presence of CL4 (~40%, respect to CL4Sc negative control) (Figure 3E).

### CL4 inhibits EGFRvIII-expressing glioblastoma cell migration and invasion

Expression of EGFRvIII increases GBM cell motility *in vitro* [33, 34] that is further enhanced in cells missing PTEN [35]. Thus, to evaluate the biological significance of the CL4-dependent EGFRvIII inhibition, we investigated whether the aptamer could affect migration and invasion of GBM cells overexpressing EGFRvIII. We addressed this question in U87MG/EGFRvIII and Gli36 GBM cells stably expressing human EGFRvIII (Gli36/EGFRvIII) (supplementary Figure S4A, left). The latter cell line, differently from U87MG, expresses PTEN (supplementary Figure S4A, right) [31, 36]. We assessed the phosphorylation of ERK1/2 and AKT proteins as read-out for monitoring the effect of CL4 in Gli36/EGFRvIII and, as shown (supplementary Figure S4B), a significant reduction not only of ERK1/2 but also of AKT activation was observed followed aptamer treatment, thus consistent with the wild-type PTEN expression in this cell line.

**Figure 4:**
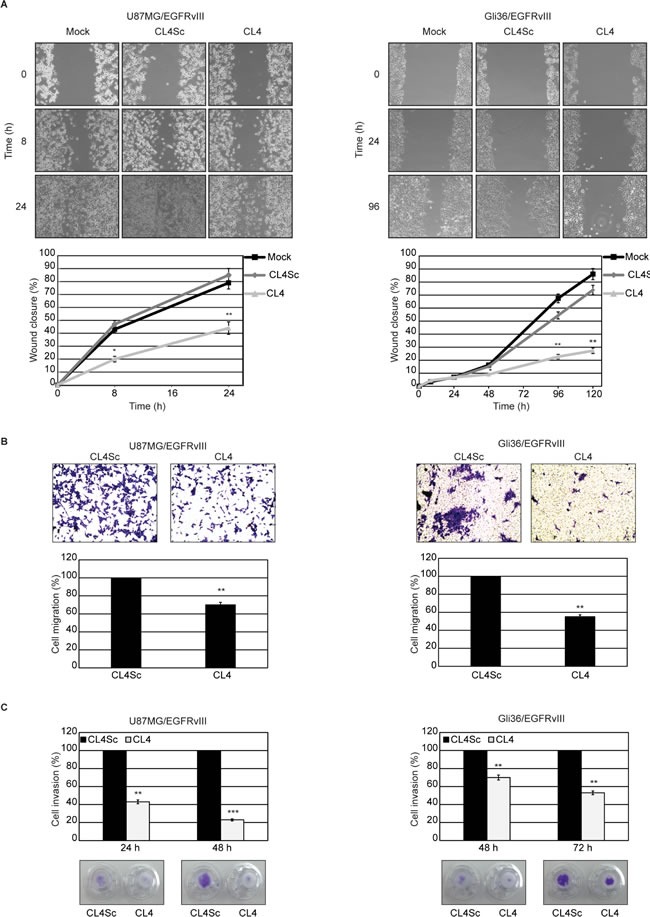
CL4 inhibits EGFRvIII-GBM cell migration and invasion **A.** Subconfluent cell monolayers were subjected to scratch assays and mock-treated or treated with CL4 or CL4Sc up to 24 (U87MG/EGFRvIII) and 120 hours (Gli36/EGFRvIII). Phase-contrast microscopy images were taken at the indicated time and the extent of wound closure was calculated. **B.** Cell motility was analyzed by transwell migration assay in the presence of CL4 or CL4Sc for 24 (U87MG/EGFRvIII) and 72 hours (Gli36/EGFRvIII). **C.** Invasion U87MG/EGFRvIII and Gli36/EGFRvIII cells through matrigel toward 10% FBS was carried out in the presence of CL4 or CL4Sc for the indicated times. **(B, C)** Data are presented as percentage of migrated or invaded cells in the presence of CL4 compared with CL4Sc control. Photographs of a representative experiment are shown. Each determination represents the average of three individual experiments and error bars represent SD. ***P < 0.001; **P < 0.01; *P < 0.05 relative to CL4Sc.

First, by using a scratch-wound assay that measures cell motility, we observed that, at 24 hours after scratch, the wound was barely visible in the plates containing U87MG/EGFRvIII cells (Figure 4A, left) whereas Gli36/EGFRvIII cells still had a wide gap that was markedly reduced at 96 hours and almost closed at 120 hours (Figure 4A, right). Thus, U87MG/EGFRvIII cells have a greater migratory activity than Gli36/EGFRvIII cells, in good agreement with previous finding that PTEN expression negatively affects glioma cell migration also in the presence of the constitutively active EGFRvIII mutant [34, 35]. Importantly, CL4 treatment of both cell lines significantly delayed the wound closure compared to mock-treated cells or cells treated with CL4Sc (Figure 4A).

Further, migration was analyzed by a “transwell migration assay” that assesses the chemotactic capacity of cells. As compared to cells treated with CL4Sc, in the presence of CL4, migration was reduced by 30% in U87MG/EGFRvIII cells and 45% in Gli36/EGFRvIII cells at 24 and 72 hours, respectively (Figure 4B). At these experimental time-points, the influence of CL4 aptamer on cell proliferation was absent (supplementary Figure S5), thus indicating that the CL4-mediated decrease in migration occurred independently of cell proliferation.

We then examined the effect of CL4 on the capacity of the cells to invade through a matrigel-coated membrane by a “transwell invasion assay”, which has been reported to mimic the whole process of cell invasion of basement membranes [37]. Using this assay, we observed that the invasion rate of U87MG/EGFRvIII and Gli36/EGFRvIII cells in the presence of CL4 treatment was significantly decreased compared with CL4Sc-treated cells (Figure 4C).

Altogether, these results show the ability of CL4 aptamer to affect GBM cell migration and invasion, also confirming the crucial role of EGFRvIII-associated signaling events for promoting cell motility.

### CL4 inhibits EGFRvIII-expressing glioblastoma cell growth

**Figure 5:**
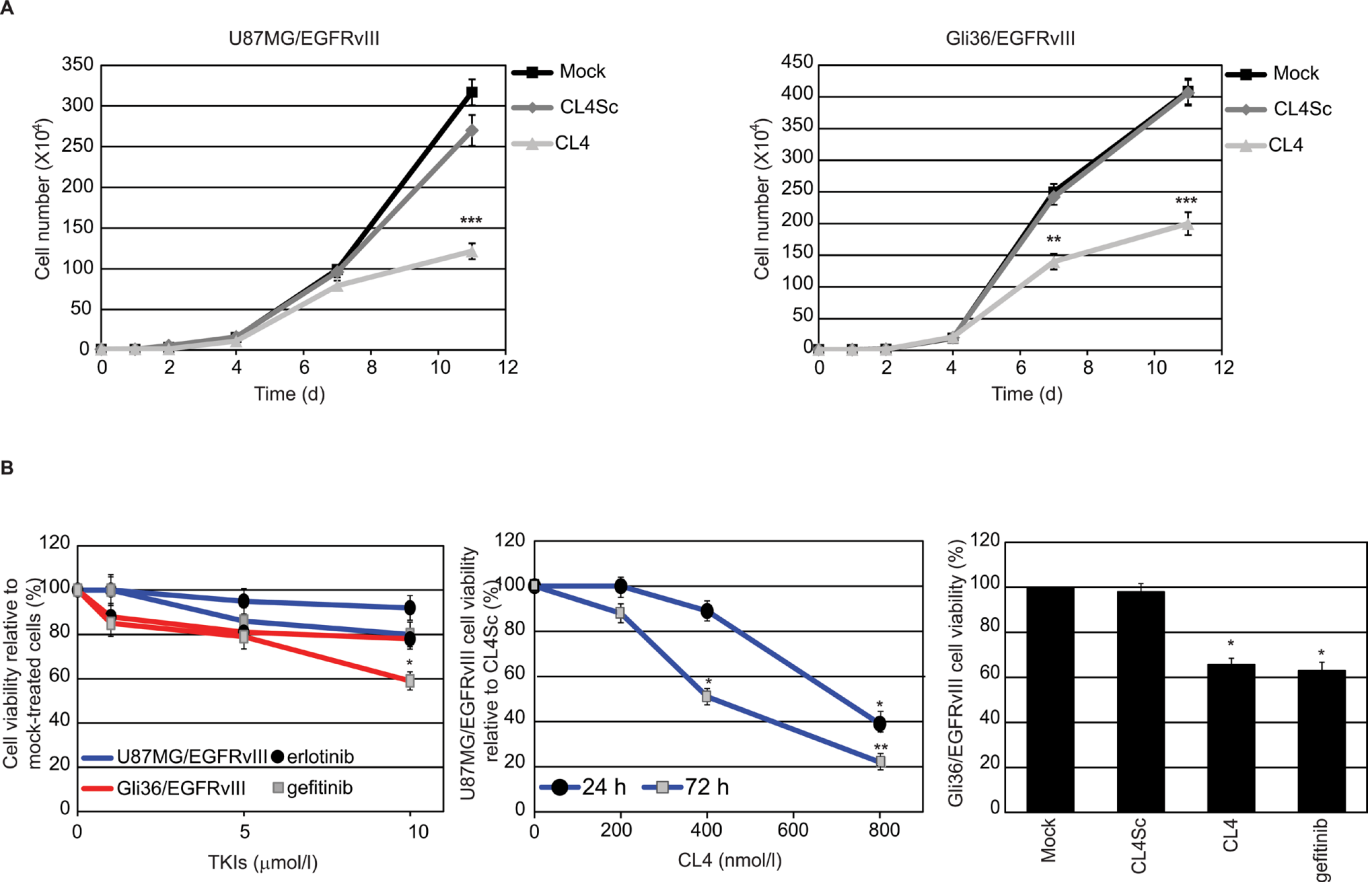
CL4 inhibits EGFRvIII-GBM cell growth **A.** U87MG/EGFRvIII (left) and Gli36/EGFRvIII (right) cells (10^4^ cells/3.5-cm plate) were either mock-treated or treated with CL4 or CL4Sc (200 nmol/l-final concentration) and counted through the Bürker chamber at the indicated time points. The aptamer treatment was renewed each 24 hours. Growth curves represent the average of three independent experiments and error bars represent SD. ***P < 0.001; **P < 0.01 relative to CL4Sc. **B.** Left, U87MG/EGFRvIII (blue line) and Gli36/EGFRvIII (red line) cells were mock-treated or treated for 72 hours with increasing amounts of erlotinib or gefitinb. Middle, U87MG/EGFRvIII cells were treated for the indicated times with increasing amounts of CL4 or CL4Sc. Right, Gli36/EGFRvIII cells were mock-treated or treated for 72 hours with 10 μmol/l gefitinib or 200 nmol/l CL4 or CL4Sc. Cell viability was analyzed and expressed as percent of viable treated cells with respect to mock-treated (left and right) or CL4Sc (middle) controls. In middle, no statistically significant variations among CL4Sc- and mock-treatment was obtained. Each determination represents the average of three individual experiments and error bars represent SD. **P < 0.01; *P < 0.05 relative to controls.

Next, we performed growth curves experiments to determine whether the long-term CL4 treatment alters the proliferation rate of EGFRvIII-expressing GBM cells. We found that the aptamer reduced the proliferation of U87MG/EGFRvIII and Gli36/EGFRvIII cells causing about 50% inhibition at day 11 with respect to cells mock-treated or treated with CL4Sc, that proliferated at comparable rates (Figure 5A).

We determined whether the aptamer was also able to reduce viability of U87MG/EGFRvIII and Gli36/EGFRvIII cells, by comparing its effects to those of erlotinib and gefitinib. As assessed by 3-(4,5-dimethylthiazol-2-yl)-2,5-diphenyltetrazolium bromide (MTT) assay, both cell lines were highly resistant to 72 hours-treatment with erlotinib and gefitinib and significant decrease in cell viability was observed only in Gli36/EGFRvIII cells treated with high concentration of gefitinib (10 μmol/l) (Figure 5B, left). As a positive control of inhibitory efficacy, we verified that the two compounds, even at the lowest doses, consistently reduced viability of non-small cell lung cancer (NSCLC) HCC827 cells that are reported to be sensitive to EGFR TKIs [38] (supplementary Figure S6). Regarding the effect of CL4, we observed that the aptamer caused a time and dose-dependent inhibition of U87MG/EGFRvIII cell viability (Figure 5B, middle). Further, almost 30% inhibition of Gli36/EGFRvIII cells viability was obtained in the presence of 72 hours-treatment with 200 nmol/l CL4 aptamer, that is comparable with that obtained with the highest gefitinib concentration tested (10 μmol/l) (Figure 5B, right).

### CL4 cooperates with an anti-PDGFRβ aptamer to inhibit proliferation of EGFRvIII-expressing GBM cells

**Figure 6:**
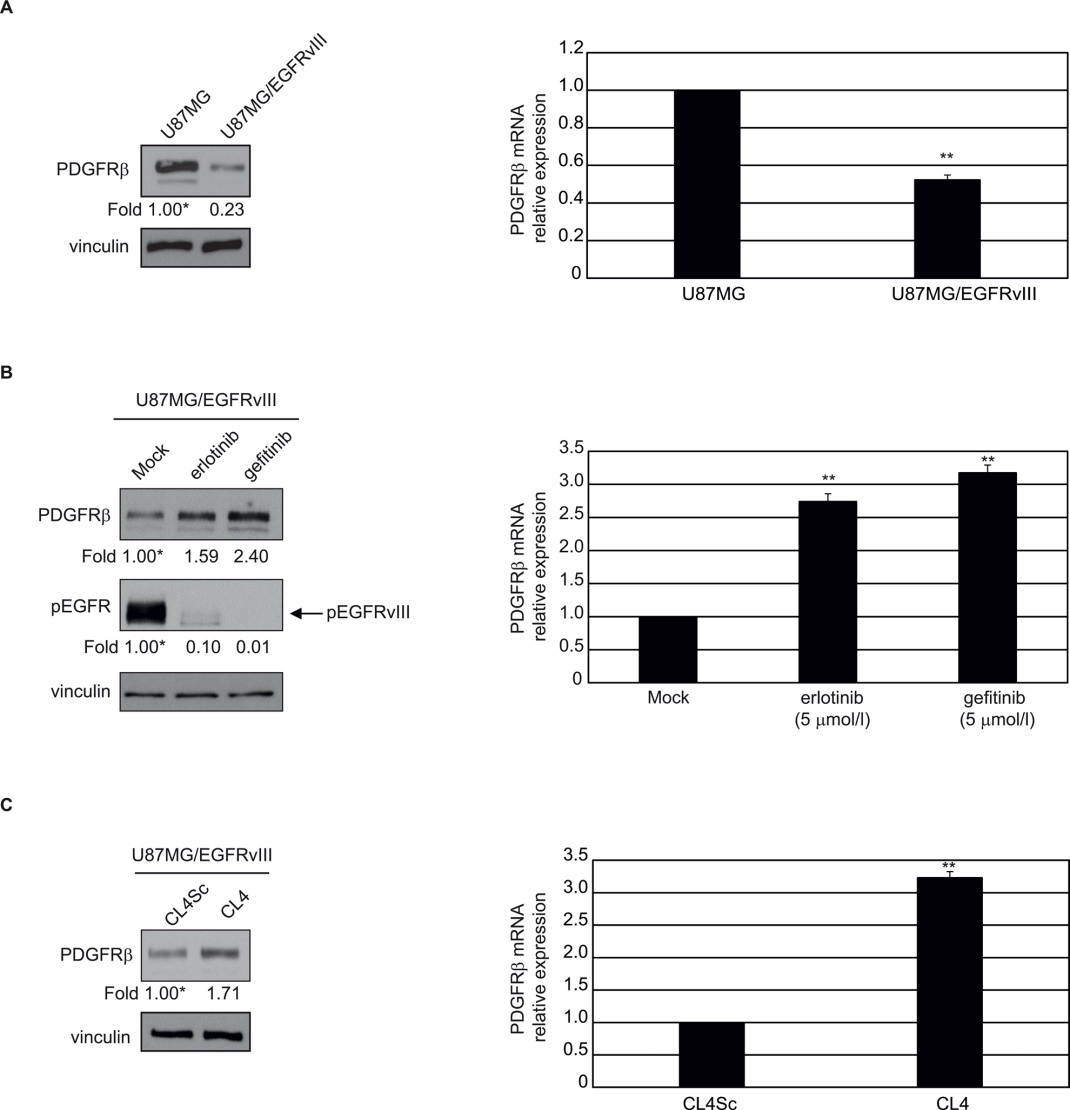
CL4-mediated inhibition of EGFRvIII induces upregulation of PDGFRβ The indicated cell lines were maintained in 2% FBS-containing medium for 24 hours in the absence **A.** and **B.** and in the presence of 5 μmol/l gefitinib or erlotinib **B.** or 200 nmol/l CL4 or CL4Sc **C.**. Cell lysates were analyzed by immunoblot with anti-PDGFRβ and anti-pEGFR antibodies, as indicated, and equal loading was confirmed by immunoblot with anti-vinculin antibody (left). Values below the blots indicate signal levels relative to each control, arbitrarily set to 1 (labeled with asterisk). PDGFRβ mRNA levels were analyzed by RT-qPCR (right). Bars depict means ± SD of three independent experiments. **P < 0.01 relative to U87MG **A.**, mock-treated **B.** or CL4Sc **C.**.

Recently, a transcriptional repressive mechanism has been described by which EGFRvIII regulates PDGFRβ expression, showing that GBMs may evade EGFR TKIs treatment by developing PDGFRβ-dependence for survival [19]. Differently from Gli36, that do not express detectable levels of PDGFRβ (not shown), U87MG are PDGFRβ-positive, and both protein (Figure 6A, left) and mRNA (Figure 6A, right) levels were markedly reduced upon stable EGFRvIII overexpression. Furthermore, in agreement with previous findings [19], inhibition of EGFRvIII signaling by 24 hours-treatment with 5 μmol/l erlotinib or gefitinib resulted in upregulation of PDGFRβ (Figure 6B). Also, when U87MG/EGFRvIII cells were treated with CL4 aptamer, under the experimental conditions (24 hours treatment at 200 nmol/l-concentration) that caused phospho-EGFRvIII inhibition (see Figure 3C), we observed an induction of PDGFRβ protein and mRNA at a comparable extent with respect to EGFR TKIs (Figure 6C).

**Figure 7:**
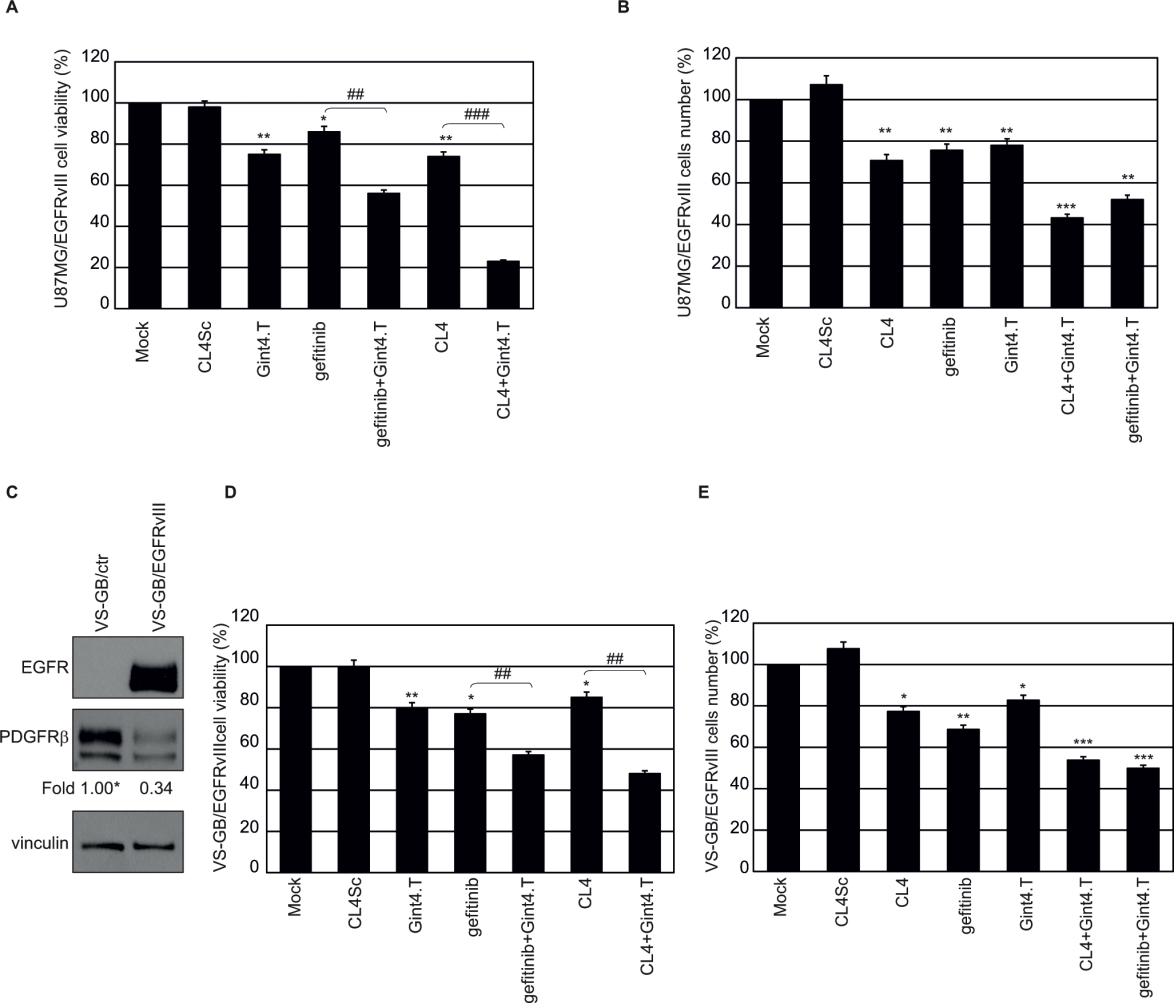
Combined inhibition of EGFRvIII and PDGFRβ in EGFRvIII-expressing GBM cells **A.** and **B.** U87MG/EGFRvIII cells (in A, 3.6 × 10^3^ cells/well on 96-well plates; in B, 10^5^ cells/3.5-cm plate) were mock-treated or treated for 24 hours with 5 μmol/l gefitinib or 200 nmol/l CL4. Then the incubation was prolonged for further 72 hours with the above treatments alone or in combination with 200 nmol/l Gint4.T, by renewing the treatment each 24 hours. As a negative control, cells were treated with 400 nmol/l CL4Sc for 96 hours. In A, cell viability was analyzed and expressed as percent of viable treated cells with respect to mock-treated cells. In B, cells were counted through the Bürker chamber and cell number with respect to mock-treated cells, was reported. **C.** Lysates from VS-GB/ctr or VS-GB/EGFRvIII cells were immunoblotted with anti-EGFR and anti-PDGFRβ antibodies. Equal loading was confirmed by immunoblot with anti-vinculin antibody. Values below the blots indicate signal levels relative to VS-GB/ctr, arbitrarily set to 1 (labeled with asterisk). **D.** and **E.** VS-GB/EGFRvIII cells were treated as in A, B and cell viability **D.** and cell number **E.** were reported as for U87MG/EGFRvIII cells. Bars depict means ± SD of three independent experiments. **P < 0.01; *P < 0.05 relative to controls (mock-treated and CL4Sc). ### *P* < 0.001; ## *P* < 0.01.

Thus, we tested whether targeting PDGFRβ by a highly specific aptamer (Gint4.T) [22] would enhance the sensitivity of U87MG/EGFRvIII cells to treatment with gefitinib or the CL4 aptamer. To this aim, cells were treated with a fixed dose of EGFR inhibitors (200 nmol/l CL4 aptamer or 5 μmol/l gefitinib) for 24 hours, to cause upregulation of PDGFRβ, and then treated for further 72 hours with CL4 or gefitinib, alone or in combination with 200 nmol/l anti-PDGFRβ Gint4.T aptamer. To test the effect of Gint4.T alone, cells were mock-treated for the first 24 hours and then treated with 200 nmol/l Gint4.T for the following 72 hours. As shown in Figure 7A, the combined targeting of EGFRvIII/EGFR and PDGFRβ led to a higher decrease in cell viability compared to single receptor blocking. The most drastic effect was observed by using the two aptamers in combination, leading to almost 77% inhibition of cell viability compared to mock-treated cells or cells treated with CL4Sc (Figure 7A).

Further, in order to distinguish whether the above treatments caused cell death or reduction of cell proliferation, we performed staining with tetramethylrhodamine, ethyl ester (TMRE) and flow cytometry analysis, staining with trypan blue, morphological analysis or immunoblot analysis of PARP cleavage. No signs of apoptosis were observed following treatment of the cells with gefitinib or CL4, alone or in combination with Gint4.T aptamer (data not shown). Instead, a consistent reduction of cell proliferation was observed reaching about 50%, in the presence of CL4 (or gefitinib) *plus* Gint4.T, compared to controls (Figure 7B).

Then, to test whether the combined blocking of EGFRvIII and PDGFRβ decreases cell proliferation also in the absence of EGFRwt expression, we used the primary GBM VS-GB/EGFRvIII cell lines. As shown, VS-GB cells endogenously express PDGFRβ, whose levels decrease in the presence of EGFRvIII overexpression (Figure 7C). Under the same treatment conditions as for U87MG/EGFRvIII cells, VS-GB/EGFRvIII cells viability was slightly decreased in the presence of CL4 aptamer as well as gefitinib. Importantly, a combination of CL4 (or gefitinib) and Gint4.T had a greater effect in inhibiting cell viability compared with either compound alone and the inhibition resulted stronger in the presence of the two aptamers (CL4 *plus* Gint4.T, 52% inhibition; gefitinib *plus* Gint4.T, 43% inhibition) (Figure 7D). Further, the co-treatment of the cells with the two aptamers reduced cell proliferation at a level similar to that observed in U87MG/EGFRvIII cells (Figure 7E).

These results emphasize the use of the two aptamers aimed at targeting both EGFRvIII and PDGFRβ receptors.

## DISCUSSION

Treatment of GBM has seen little improvement in the last 20 years despite targeted therapy and progress in surgery, radiation delivery and chemotherapy. Innovative therapeutic approaches are therefore needed to combat this highly lethal cancer. Therapies targeting EGFR, one of the most important oncoprotein involved in human cancer, while showing efficacy in other tumors, have not delivered long term benefits to GBM patients. EGFRvIII, the truncated extracellular mutant of the EGFR, is the most common EGFR mutant found in GBM and usually co-exists with EGFRwt. Increased EGFRvIII expression can play major roles in promoting tumor cell proliferation, migration, invasiveness and resistance to treatment [39, 40]. In this study, by using RT-qPCR, flow cytometry and immunofluorescence methods, we prove that the CL4 nuclease-stabilized RNA aptamer, previously generated as a neutralizing ligand for human EGFRwt [21, 22], is able to bind to EGFRvIII despite the lack of a portion of the extracellular region in EGFRvIII. Accordingly, crosslinking experiments among the soluble extracellular domain of EGFRwt and the aptamer revealed that the interaction between CL4 and EGFR is with domain IV of the receptor, which is still present in EGFRvIII. More detailed investigation of the interaction of CL4 to both variants, in a purified form, will help in defining whether the extracellular part of the protein, that is present in EGFRwt but not in EGFRvIII, could have a role in modulating the binding affinity and specificity.

It has been clearly shown that EGFRvIII monomers can pair and form disulfide-bonded homodimers that enhance receptor oncogenicity [27, 28]. Further, chemically induced dimerization of a chimeric EGFRvIII in U87MG cells causes an increase of EGFRvIII and ERK1/2 phosphorylation levels, strengthens receptor tumorigenic signal and reduces survival of U87MG intracranial xenograft-bearing mice [29]. Moreover, it has been reported that gefitinib and other quinazoline TKIs, while inhibiting multiple EGFRvIII signaling pathways at high and repeated doses, when used at low concentrations, cause augmented homodimerization of the receptor, increased signaling to ERK1/2 and stimulation of proliferation and anchorage-independent growth of EGFRvIII-expressing GBM cells [41, 42]. In agreement with these observations, we show that the treatment of EGFRvIII-expressing U87MG cells with CL4 strongly inhibits the formation of high-order EGFRvIII active complexes that is accompanied by a consistent reduction of the phosphorylation of ERK1/2 and STAT3 downstream signaling effectors. Conversely, it does not affect AKT activity in U87MG/EGFRvIII cells. This is likely due to a lack of regulation in the PI3K pathway in these PTEN negative cells, since when applied to GBM PTEN-positive Gli36/EGFRvIII cells, the aptamer was able to reduce AKT phosphorylation.

Given the important contributing role of ERK1/2 and STAT3 pathways to the malignant phenotype of EGFRvIII-expressing GBM cells [15, 33, 42-44], we looked at the effects of CL4 on migration, invasion and proliferation of U87MG/EGFRvIII and Gli36/EGFRvIII cells. In agreement with previous reports indicating that the expression of EGFRvIII in GBM cells promotes cell migration, confers a significant growth advantage and enhance tumorigenic activity also in the context of EGFRwt expression [33, 45-47], we found that U87MG/EGFRvIII cells had a higher proliferation rate and ability to migrate (in both “scratch” tests and “transwell” assays) compared to mock-transfected U87MG cells (data not shown). Importantly, when applied to U87MG/EGFRvIII and Gli36/EGFRvIII cells, CL4 aptamer significantly reduced cell migration, invasion and proliferation.

Recent reports indicate that targeting EGFRvIII causes the occurrence of parallel signaling pathways that render GBM cells resistant to EGFR inhibitors [19, 48]. It has been shown that EGFRvIII suppresses PDGFRβ expression in GBM and that EGFR inhibitors, by de-repressing PDGFRβ, render GBM dependent on PDGFRβ signaling for growth and survival [19]. Accordingly, silencing PDGFRβ significantly attenuated the growth of tumors from U87MG cells expressing kinase dead-EGFRvIII subcutaneously implanted in mice [19]. Moreover, we found that U87MG/EGFRvIII cells resulted highly resistant to erlotinib and gefitinib and significantly inhibited in cell viability upon CL4 treatment only at high doses or by prolonging the incubation times. Despite the poor effect on cell viability, we found that incubating the cells with 200 nmol/l CL4, as well as with 5 μmol/l gefitinib or erlotinib, causes a significant fold increase of PDGFRβ. Altogether these observations prompt us to test aptamer-based combined treatments aimed at blocking both EGFRvIII and PDGFRβ signaling.

We have recently generated an aptamer, named Gint4.T, which binds to PDGFRβ and causes a strong inhibition of PDGF-BB ligand-dependent receptor activation in cell lines and primary cultures of GBM cells [22]. In addition, we have shown that, by targeting EGFRwt and PDGFRβ, CL4 and Gint4.T synergize at slowing the growth of xenografts from U87MG cells [22]. Here, we demonstrated that the combined treatment of U87MG/EGFRvIII cells with CL4 and Gint4.T aptamers led to a consistent higher inhibition of cell growth with respect to each aptamer alone, without induction of cell death. Further, the anti-PDGFRβ aptamer also restored the cell sensibility to gefitinib treatment, thus confirming a greater advantage of targeting both EGFR/EGFRvIII and PDGFRβ over single receptor inhibition.

The expression of EGFRvIII is usually associated with EGFRwt amplification in GBM clinical samples, thus highlighting the potential benefit of the CL4 aptamer targeting both the wild-type and the mutated receptor. However, the efficacy of CL4, used in combination with the anti-PDGFRβ aptamer, was also extended to a PDGFRβ-positive GBM primary cell line lacking endogenous EGFRwt and engineered to express EGFRvIII (VS-GB/EGFRvIII), thus indicating the relevance of blocking both EGFRvIII and PDGFRβ also in the absence of the wild-type receptor.

Future studies in animal models will help to determine whether this is a viable strategy for GBM treatment.

Further, it has been shown that in GBM cells, EGFRvIII transactivates other RTKs, including the hepatocyte growth factor (HGF) receptor (MET) and co-targeting of these RTKs has a potent antitumor efficacy, thus suggesting a potential strategy for treating EGFRvIII-expressing gliomas [49]. Our present findings showing the ability of CL4 aptamer to inhibit EGFRvIII and the value of aptamer-based inhibition of EGFR and PDGFRβ, strongly encourage to further test whether the aptamer will reveal effective in interfering with EGFRvIII-dependent crosstalk with other RTKs, in addition to PDGFRβ.

Due to the essential advantages of oligonucleotide aptamers such as their lack of immunogenicity, high stability, low-cost and rapid production with high batch fidelity [50-53], CL4 and Gint4.T may represent an attractive alternative over exiting anti-EGFR and anti-PDGFRβ therapeutics and will help to expand current limited management options for the treatment of GBM.

## MATERIALS AND METHODS

### Cell lines and transfection

Growth conditions for human GBM U87MG and mouse NIH3T3 (American Type Culture Collection, Manassas, VA) cell lines were previously reported [21, 22]. VS-GB Primary cell culture from GBM specimens was derived and grown as described previously [22]. VS-GB/EGFRvIII and NIH/EGFRvIII were obtained from VS-GB and NIH3T3 cells, respectively, stably transfected with EGFRvIII plasmid (kindly provided by K. Latha, Anderson Cancer Center) by Lipofectamine 2000 (Invitrogen, Carlsbad, CA), selected in 800 μg/ml Zeocin (Invitrogen) and maintained in DMEM-F12 with 10% fetal bovine serum (FBS) (VS-GB/EGFRvIII) or DMEM with 10% bovine calf serum (NIH/EGFRvIII) in 95% air/5% CO2 atmosphere at 37°C. NIH/EGFRwt were obtained from NIH3T3 cells stably transfected with EGFRwt plasmid (Addgene, kindly provided by M. Meyerson [54]) by Lipofectamine 2000 (Invitrogen) and selected in 10 μg/ml Puromycin dihydrochloride (Sigma-Aldrich, St. Louis, MO). Human GBM U87MG/EGFRvIII, Gli36 and Gli36/EGFRvIII cells were previously described [55, 56]. Human NSCLC HCC827 (American Type Culture Collection) were grown in RPMI-1640 supplemented with 10% FBS.

### Aptamers and treatments

2′F-Py RNA aptamers (CL4, Gint4.T, CL4Sc and 5′ FAM-labeled CL4) were synthesized by TriLink Biotechnologies and purchased from Tebu-bio srl (Magenta, Milan, Italy).

CL4:5′GCCUUAGUAACGUGCUUUGAUGUCGAUUCGACAGGAGGC3′.

CL4Sc, used as a negative control:5′UUCGUACCGGGUAGGUUGGCUUGCACAUAGAACGUGUCA3′.

Gint4.T:5′UGUCGUGGGGCAUCGAGUAAAUGCAAUUCGACA3′.

Before each treatment, the aptamers were subjected to a short denaturation-renaturation step (85°C for 5 minutes, on ice for 2 minutes, 37°C for 10 minutes). For cell incubation longer than 24 hours, the treatment was renewed each day and the RNA concentration was determined to ensure the continuous presence of at least 200 nmol/l concentration, taking into account the 6 hours-half-life of the aptamer in 10% serum.

### Protein extraction and Immunoblot

For protein extraction, cells were lysed in lysis buffer containing 50 mM Hepes (pH 7.5), 150 mM NaCl, 1% glycerol, 1% Triton X-100, 1.5 mM MgCl2, 5 mM EGTA, 1 mM Na3VO4, supplemented with protease inhibitors (Roche Diagnostics, Indianapolis, IN). Protein concentration was determined by the Bradford assay using Bovine serum albumin (BSA) (Panreac AppliChem, Darmstadt, Germany) as the standard. SDS-PAGE analysis, under reducing and non-reducing conditions, was carried out according to Laemmli in the presence and absence of 2-mercaptoethanol (Sigma-Aldrich), respectively. Gels were electroblotted into polyvinylidene difluoride membranes (Millipore Co., Bedford, MA) and filters were probed with the indicated primary antibodies: anti-phospho-EGFR (Tyr1068, indicated as pEGFR), anti-EGFR (intracellular), anti-phospho44/42 MAPK (D13.14.4E, indicated as pERK), anti-phospho-AKT (Ser473, indicated as pAKT), anti-AKT, anti-phospho-STAT3 (indicated as pSTAT3), anti-STAT3, anti-PDGFRβ (Cell Signaling Technology Inc., Danvers, MA); anti-ERK1 (C-16), anti-α-tubulin (TU-02), anti-vinculin (N-19) (Santa Cruz Biotechnology, Santa Cruz, CA); anti-PTEN (Y184; Abcam, Cambridge, MA).

Densitometric analyses were performed on at least two different expositions to assure the linearity of each acquisition using ImageJ (v1.46r). Blots shown are representative of at least three independent experiments.

### Binding of CL4 aptamer to the cells

#### Binding assay by RT-qPCR

Binding was performed as described previously [57]. Briefly, the day before the treatment, cells were seeded in 3.5-cm plates (2 × 10^5^ cells/plate). Cells were incubated with 200 nmol/l and 400 nmol/l CL4 or CL4Sc negative control, for 15 minutes at 37°C in the presence of 100 μg/ml polyinosine (Sigma-Aldrich) as a nonspecific competitor. Unbound RNA was removed by washing (3 × 500 μl of ice-cold Dulbecco's Phosphate-Buffered Saline (DPBS) and bound RNA was recovered by TRIzol (Invitrogen) containing 0.5 pmoles/ml of starting RNA library [55], used as a reference control and here indicated as random pool. Recovered RNA was reverse transcribed (RT) using M-MuLV Reverse Transcriptase (Roche Diagnostics) and specific 3′ primers. The primers for the RT step were: CL4 3′ primer, 5′-GCCTCCTGTCGAATCGA-3′; CL4Sc 3′ primer, 5′-TGACACGTTCTATGTGCA-3′; random pool 3′ primer, 5′-GCCTGTTGTGAGCCTCCTGTCGAA-3′. The RT protocol was as follows: the recovered RNA and the primers were heated at 65°C for 5 minutes, annealed at 22°C for 5 minutes and extended at 42°C for 30 minutes followed by an extension at 48°C for 30 minutes and enzyme inactivation at 95°C for 5 minutes. The product from the RT reaction was PCR amplified with iQ SYBR Green Supermix (Bio-Rad, Hercules, CA) in the presence of CL4, CL4Sc or random pool 5′ and 3′ primers. The 3′ primers were as in the RT step and the 5′ primers were: CL4 5′ primer,5′-TAATACGACTCACTATAGGGGCCTTAGTAACG-3′; CL4Sc 5′ primer,5′-TAATACGACTCACTATAGGGTTCGTACCGGGT-3′; random pool 5′ primer,5′-TAATACGACTCACTATAGGGAGACAAGAATAAACGCTCAA-3′. The qPCR protocol was as follows: the RT product was heated at 95°C for 2 minutes, followed by 40 cycles of heating at 95°C for 30 seconds, annealing at 55°C for 30 seconds, and extending at 60°C for 30 seconds. A melt curve stage by heating at 60-95°C was performed.

Reactions were all done in 25 μL volume in triplicate. Data were normalized to the random pool reference control and to the cell number, as determined by counting cells cultured in conjunction with each experiment.

#### Binding assay by confocal microscopy

NIH/ctr and NIH/EGFRvIII were seeded on coverslip in 24-well (10^5^ cells/well) in DMEM (PAA, E15-009) supplemented with 10% FBS (PAA, A15-151), 2 mM L-glutamine and 100 units/ml penicillin-streptomycin at 37°C in an atmosphere of 5% CO2/air. After 24 hours cells were treated with 2.5 μmol/l FAM-labelled CL4 in DMEM without FBS for the indicated time. Then, cells were washed three times in DPBS and fixed in DPBS/PFA 4% for 20 minutes. For binding assay, unpermeabilized cells were subjected to blocking in DPBS/BSA 0.5% for 1 hour, and incubated with anti-EGFR antibody (AF231, R&D system, Minneapolis, MN). After three washes in DPBS, cells were incubated with anti-goat Alexa Fluor 647 conjugated antibody (A21447, Life Technologies Italia, Monza, Italy). Finally, after three further washes in DPBS, cells were incubated with 1.5 μM 4′,6-Diamidino-2-phenylindole (DAPI, D9542, Sigma-Aldrich) and mounted with Fluorescence mounting medium (S3023, Dako Italia, Milan, Italy).

For endosome internalization assay, fixed cells were permeabilized with DPBS/Triton X-100 0.2% for 5 minutes and then subjected to blocking in DPBS/BSA 0.5% for 1 hour. Cells were incubated with anti-EGFR antibody and anti-EEA1 (ab2900, Abcam), washed three times in DPBS, and incubated with anti-goat Alexa Fluor 647 conjugated and anti-rabbit Alexa Fluor 555 (A31572, Life Technologies Italia). Finally, after three further washes in DPBS, cells were incubated with 1.5 μM DAPI and mounted with Fluorescence mounting medium.

Samples were visualized on a TSC SP5 confocal microscope (Leica) installed on an inverted LEICA DMI 6000CS microscope and equipped with an oil immersion PlanApo 63X 1.4 NA objective. Images were acquired using the LAS AF acquisition software (Leica).

#### Binding assay by flow cytometry

U87MG or U87MG/EGFRvIII cells were trypsinized and washed twice with 500 μl DPBS. Cells (2 × 10^5^) were incubated in the absence or in the presence of 2 μmol/l FAM-labelled CL4 in 100 μl DPBS at 37°C for 30 minutes. Cells were washed twice with 500 μl DPBS, suspended in 400 μl DPBS and analyzed by flow cytometry. The fluorescence was determined with a Flow Cytometer (BD Accuri™ C6) by counting 20.000 events.

### UV induced cross-linking experiments and MALDI-TOF mass spectrometry

Cross-linking experiments were carried out by irradiating samples with a UV lamp (Spectronics Corporation) at 254 nm. In a typical experiment, an aliquot of the EGFR ectodomain protein (R&D Systems) was combined with the CL4 aptamer in a 1:1 molar ratio in 50 mM Tris-HCl buffer, pH 7.5. The sample was then irradiated at 254 nm for 20 minutes at 25°C at a distance of 7 cm. The cross-linked product was then digested with trypsin [enzyme/substrate ratio, 1:100 (w/w)] for 6 hours at 25°C in the same buffer, and enzymatic hydrolysis was carried out with T1 ribonuclease (300 units at 37°C for 30 minutes). The resulting peptide mixture was then directly analyzed by MALDI-MS.

Positive Reflectron MALDI spectra were recorded on a Voyager DE STR instrument (Applied Biosystems, Framingham, MA). The MALDI matrix was prepared by dissolving α-cyano-hydroxycinnamic powder in 70% acetonitrile and 30% citric acid 50 mM. Typically 1 μl of matrix was applied to the metallic sample plate and 1 μl of analyte was then added. The mixture thus obtained was then dried at room temperature. Acceleration and reflector voltages were set up as follows: target voltage at 20 kV, first grid at 65% of target voltage, delayed extraction at 400 ns to obtain the best signal-to-noise ratios and the best possible isotopic resolution. Mass calibration was performed using external peptide standards purchased from Applied Biosystems. Each spectrum represents the sum of 3,000 laser pulses from randomly chosen spots per sample position. Raw data were analyzed using the computer software provided by the manufacturers and are reported as monoisotopic masses.

### Cell migration and invasion

For wound healing assay, U87MG/EGFRvIII and Gli36/EGFRvIII cells were plated in six-well plates and grown to subconfluence. The assay was performed as previously described [22], in the absence or in the presence of 200 nmol/l CL4 or CL4Sc. The distance between the two edges of the scratch was measured by ImageJ (v1.46r) in three areas (top, middle and bottom), the average distance was quantified and the extent of wound closure was determined as follows: wound closure (%) = 1-(wound width tx/wound width t0) x 100. Each experiment was triplicated and performed three times independently.

For transwell migration assay, U87MG/EGFRvIII and Gli36/EGFRvIII cells were serum-starved overnight, in the presence of 200 nmol/l CL4 or CL4Sc, and then trypsinized, re-suspended in DMEM serum free, and counted. Cells (5 × 10^4^ in 100 μl serum-free medium per well) were seeded into the upper chamber of a 24-well transwell (Transwell filters 8 μm pore size; Corning Incorporate, Corning, NY) in the presence of 200 nmol/l CL4 or CL4Sc. The lower chamber was filled with 600 μl of medium containing 5% FBS, as inducer of migration.

For invasion assays transwell filters were coated with Matrigel (BD Biosciences, NJ) diluted 1:4 in serum-free medium and left 45 minutes in incubator at 37°C. Then the assay was performed as the migratory assay except that an higher number of cells (1 × 10^5^) were exposed to 10% FBS.

After incubation at 37°C in humidified 5% CO_2_ for indicated times, cells were visualized by staining with 0.1% crystal violet in 25% methanol and photographed. Stained cells were lysed in 1% SDS and absorbance at 595 nm was measured in a microplate reader. Each experiment was triplicated and performed three times independently.

### Cell viability

Cell viability was assessed with CellTiter 96 AQueous One Solution Cell Proliferation Assay (Promega BioSciences Inc., San Luis Obispo, CA) according to the manufacturer's instructions. In each assay, cells were plated in 96-well plates (3.6 × 10^3^ cells/well). To assess cell viability in the presence of EGFR-TKIs, erlotinib (Cell Signaling) and gefitinib (LC Laboratories, Woburn, MA) were used, as indicated.

### Real-Time PCR

RNA was extracted by TRizol (Invitrogen) according to the manufacturer's instructions. 1 μg total RNA was reverse transcribed with iScript cDNA Synthesis Kit (Bio-Rad) and the resulting cDNA fragments were amplified by using iQ SYBR Green supermix (Bio-Rad). Primers used were: PDGFRβ, Fwd 5′-AGGACACGCAGGAGGTCAT-3′, Rev 5′-TTCTGCCAAAGCATGATGAG-3′; EGFRwt, Fwd 5′-TCCTTGGGAATTTGGAAATT-3′, Rev 5′-GGCATAGGAATTTTCGTAGTACAT-3′; β-actin, Fwd 5′-CAAGAGATGGCCACGGCTGCT-3′, Rev 5′-TCCTTCTGCATCCTGTCGGCA-3′. Relative mRNA quantization was performed by using the ΔΔCt method applying the equation 2^− ΔΔCt^.

### Statistics

Statistical values were defined using GraphPad Prism version 6.00 for Windows by *t*-test. *P* value < 0.05 was considered significant.

## SUPPLEMENTARY MATERIAL FIGURES


