# Supplementary material for: Aptamer targeting EGFRvIII mutant hampers its constitutive autophosphorylation and affects migration, invasion and proliferation of glioblastoma cells
Source: Oncotarget. 2015 Oct 10;6(35):37570–87. doi: 10.18632/oncotarget.6066 (PMC4741949; doi:10.18632/oncotarget.6066)
Supplement: Supplementary file 1 [file oncotarget-06-37570-s001.pdf]

Aptamer targeting EGFRvIII mutant hampers its constitutive autophosphorylation and affects migration, invasion and proliferation of glioblastoma cells

Supplementary Material

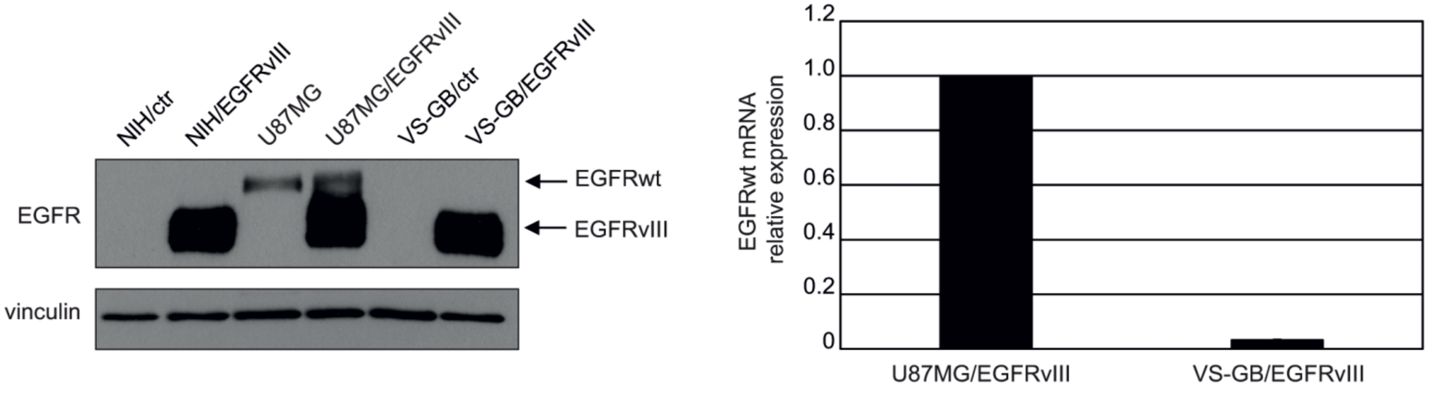

Figure S1: EGFRvIII expression in transfected cell lines. Left, lysates from NIH/EGFRvIII, U87MG/EGFRvIII and VS-GB/EGFRvIII cells and vector-transfected control cells were immunoblotted with anti-EGFR antibody. Equal loading was confirmed by immunoblot with anti-vinculin antibody. Endogenous EGFRwt of 170 kDa and exogenous EGFRvIII of 140-155 kDa are indicated by the arrow. Right, EGFRwt mRNA levels were analyzed by RT-qPCR and reported as relative to U87MG/EGFRvIII cells.

A

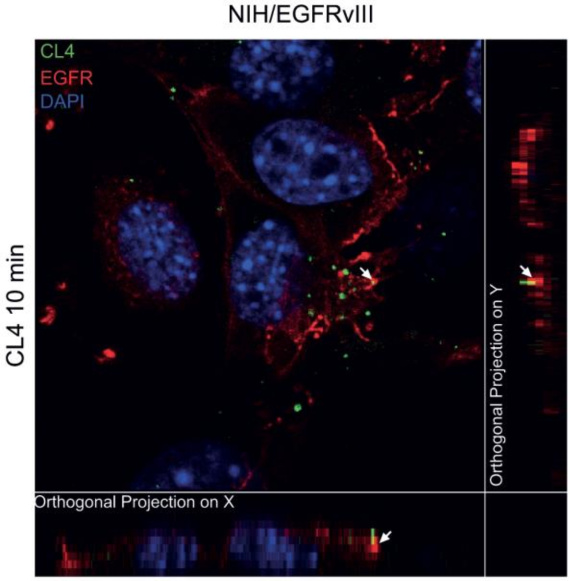

B

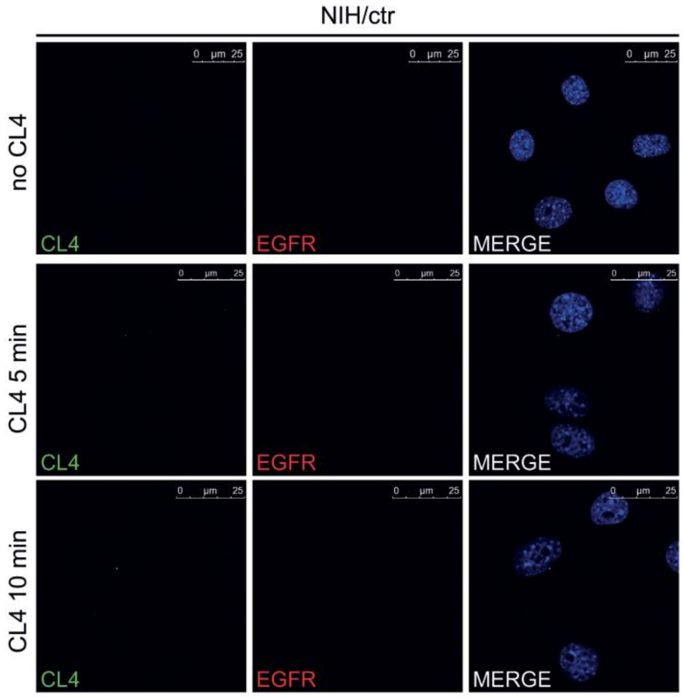

Figure S2: CL4 colocalization with EGFRvIII. (**A**) NIH/EGFRvIII cells were incubated with 2.5 µmol/l FAM-labelled CL4 for 10 minutes and then fixed and labelled with anti - EGFR antibody without permeabilization. A single focus "XY" from a Z-stack is shown indicating the plane in with CL4 and EGFRvIII colocalization occurred. Orthogonal projections of X and Y axis are also shown to highlight that colocalization occurred at membrane level. White arrows indicate the colocalization punctum between CL4 and EGFRvIII. (**B**) NIH/ctr cells were incubated with 2.5 µmol/l FAM-labelled CL4 for 5 and 10 minutes. Cells were fixed and labelled with anti - EGFR antibody without permeabilization. CL4, EGFRvIII and nuclei are visualized in green, red and blue, respectively.

A

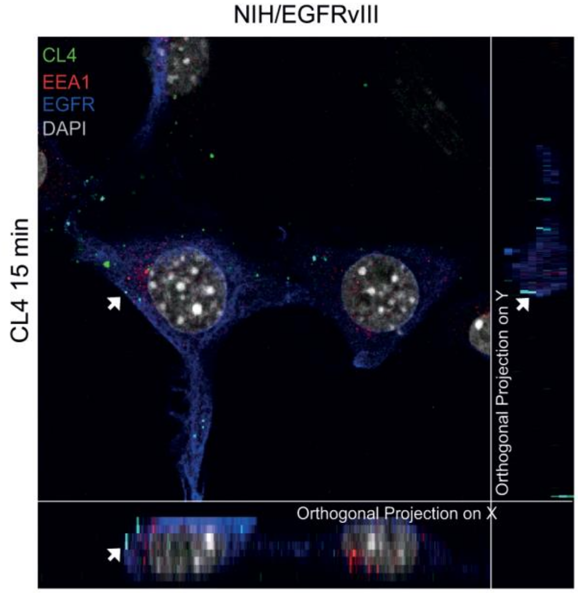

B

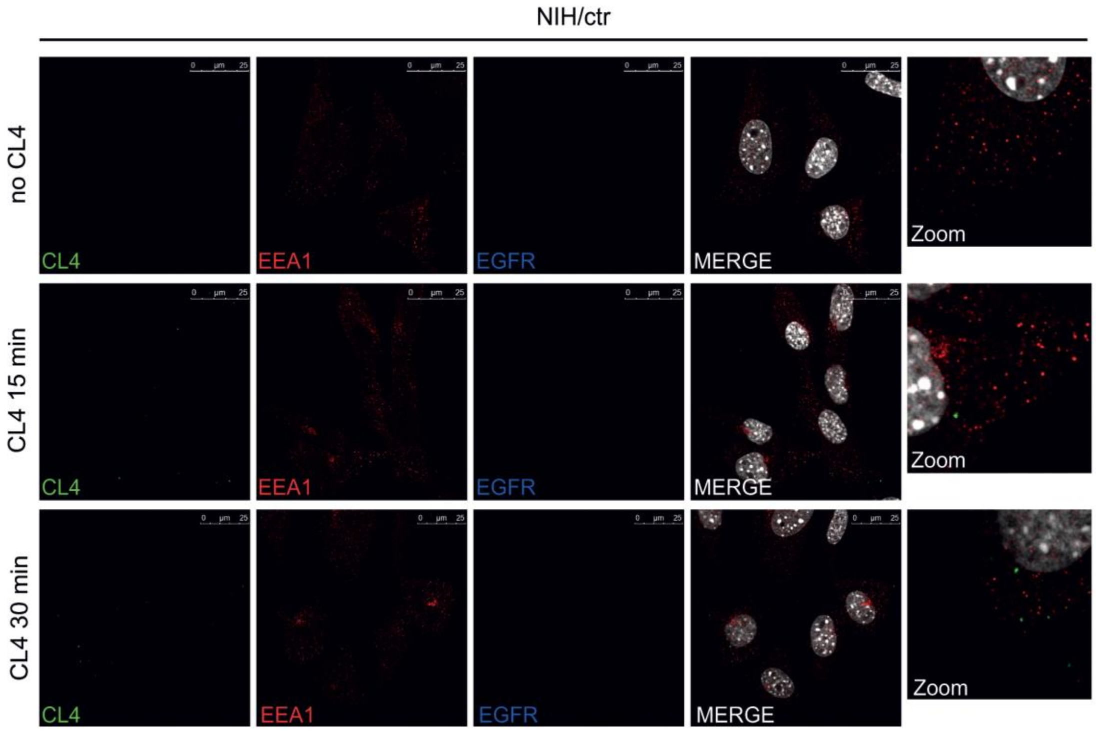

Figure S3: CL4 EGFRvIII - bound colocalization with endosome compartment . (**A**) NIH/EGFRvIII cells were incubated with 2.5 μmol/l FAM-labelled CL4, as reported in the legend to Figure 1C . Cells were fixed, permeabilized and labelled with anti-EGFR and anti-EEA1 antibodies. A single focus "XY" from a Z-stack is shown indicating the plane in with CL4 EGFRvIII - bound colocalizes with EEA1 at 15 minutes incubation. Orthogonal projections of X and Y axis are also shown to highlight colocalization. White arrow indicates one of the colocalization puncta between CL4 EGFRvIII - bound colocalized with EEA1 as highlighted in the orthogonal projections . (**B**) NIH/ctr, were incubated with 2.5 μmol/l FAM-labelled CL4 for 15 and 30 minutes, fixed, permeabilized and labelled with anti-EGFR and anti-EEA1 antibodies. CL4, EEA1, EGFRvIII and nuclei are visualized in green, red, blue and grey, respectively.

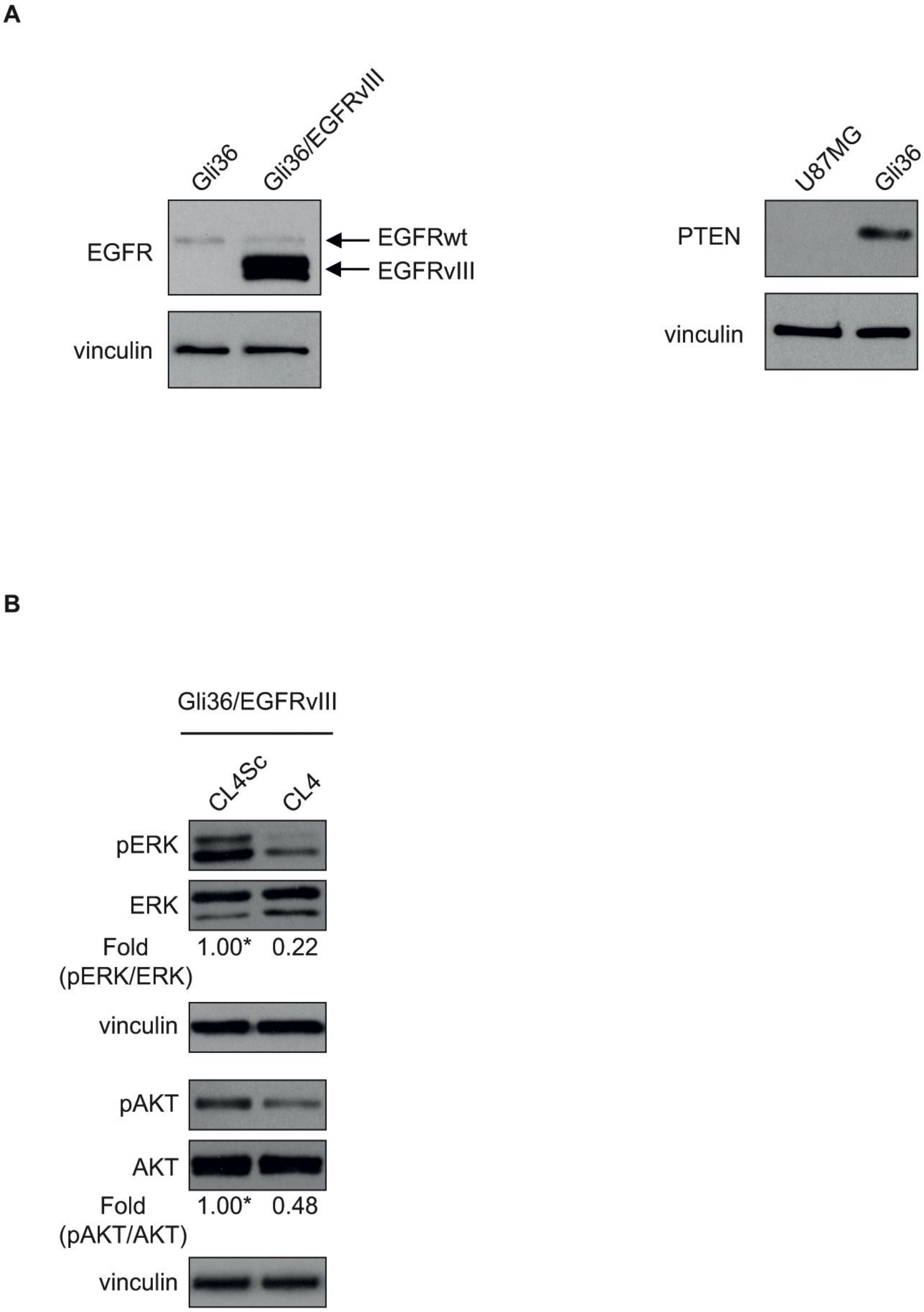

Figure S4: CL4 inhibits ERK and AKT activation in Gli36/EGFRvIII cells. (**A**) Lysates from the indicated cell lines were immunoblotted with anti-EGFR (left) or PTEN (right) antibody. (**B**) Lysates from Gli36/EGFRvIII cells treated with 200 nmol/l CL4 or CL4Sc for 30 minutes were immunoblotted with anti-pERK and anti-pAKT antibodies, as indicated. Filters were stripped and reprobed with anti-ERK and anti-AKT antibodies. A vinculin loading control is included. Values below the blot indicate signal levels reported as relative to CL4Sc, arbitrarily set to 1 (labeled with asterisk).

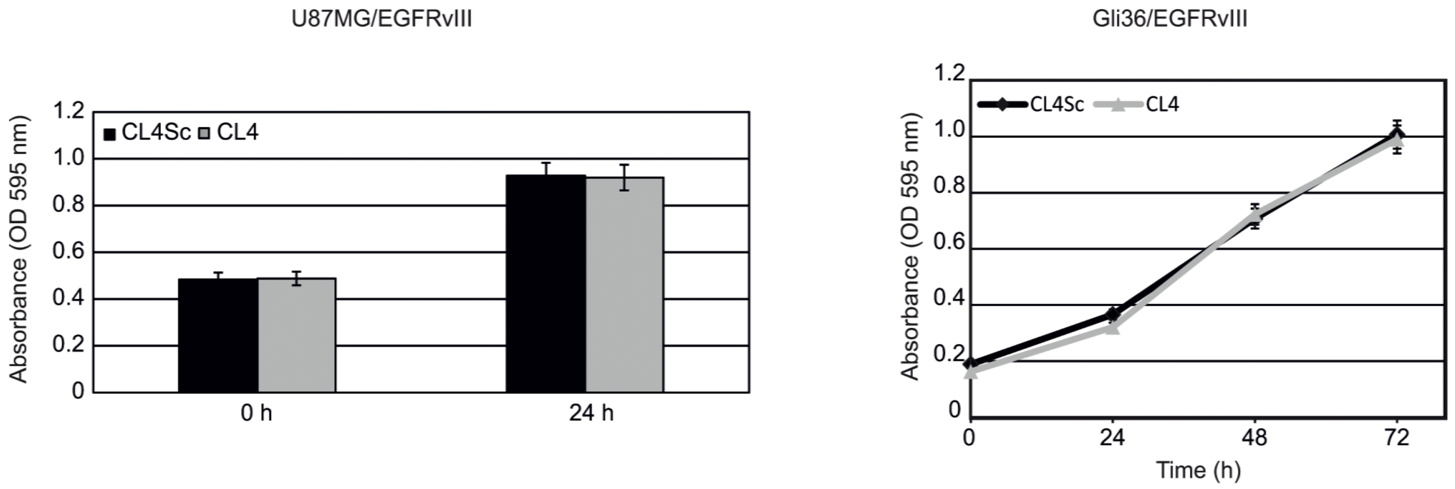

Figure S5: Proliferation rate in U87MG/EGFRvIII and Gli36/EGFRvIII cells after seeding. The indicated cell lines were serum-starved overnight in the presence of 200 nmol/l CL4 or CL4Sc and plated (5 x 10^4 cells) in each well of a 24-well plate in the presence of the above treatments. At the indicated time points, cells were stained with crystal violet. Mean values ± SD (*n* = 3) of absorbance at 595 nm of the stained cells are reported.

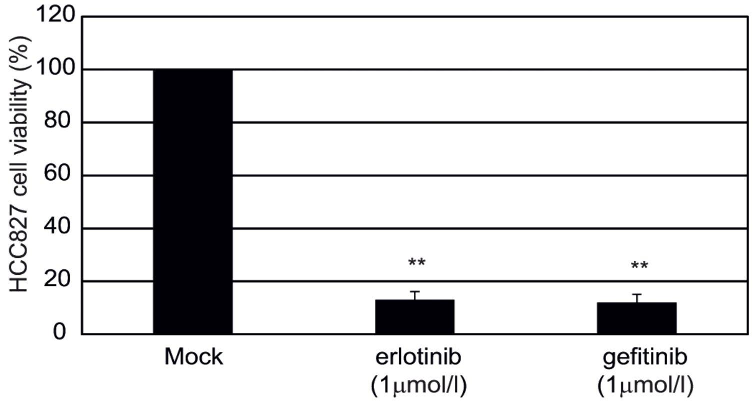

Figure S6 : HCC827 cells are sensitive to EGFR - TKIs. HCC827 cells were mock - treated or treated for 72 hours with 1 µmol/l erlotinib or gefitinb. Cell viability was analyzed and expressed as percent of viable treated cells with respect to mock - treated cells. Bars depict mean ± SD ($n = 3$). $^{**}P < 0.01$ relative to mock-treated.
